# Silkworms Can be Used as an Animal Model to Screen and Evaluate Gouty Therapeutic Drugs

**DOI:** 10.1673/031.012.0401

**Published:** 2012-01-22

**Authors:** Xiaoli Zhang, Renyu Xue, Guangli Cao, Zhonghua Pan, Xiaojian Zheng, Chengliang Gong

**Affiliations:** ^1^ Pre-clinical Medical and Biological Science College, Soochow University, Suzhou China; ^2^ National Engineering Laboratory for Modern Silk, Soochow University Suzhou, China; ^#^ These authors contributed equally to this work.

**Keywords:** allopurinol, gout, sodium bicarbonate, urate oxidase

## Abstract

In the past few decades, the mouse has been used as a mammalian model for hyperuricemia and gout, which has increased not only in prevalence, but also in clinical complexity, accentuated in part by a dearth of novel advances in treatments for hyperuricemia and gouty arthritis. However, the use of mice for the development of gouty therapeutic drugs creates a number of problems. Thus, identification and evaluation of the therapeutic effects of chemicals in an alternative animal model is desirable. In the present study, the effects of gouty therapeutic drugs on lowering the content of uric acid and inhibiting activity of xanthine oxidase were evaluated by using a silkworm model, *Bombyx mori* L. (Lepidoptera: Bombycidae). The results showed that the effectiveness of oral administration of various gouty therapeutic drugs to 5^th^ instar silkworms is consistent with results for human. The activity of xanthine oxidase of silkworm treated with allopurinol was lower, and declined in a dose-dependent manner compared with control silkworms, while sodium bicarbonate failed at inhibiting the activity of xanthine oxidase. The concentration of uric acid in the both hemolymph and fat body declined by 90 and 95% at six days post-administration with 25 mg/mL of allopurinol, respectively (*p* < 0.01), while the concentration of uric acid in both the hemolymph and fat body also declined by 81 and 95% at six days post-administration with 25 mg/mL of sodium bicarbonate, respectively (*p* < 0.01). Moreover, the epidermis of silkworm treated with allopurinol or sodium bicarbonate became transparent compared with the negative control group. These results suggest that silkworm larva can be used as an animal model for screening and evaluation of gouty therapeutic drugs.

## Introduction

Gout is a painful disease that is mainly caused by the deposition of monosodium urate crystals in joints. Elevated serum urate levels are recognized as leading to gouty arthritis, tophi formation, hyperuricemia, uric acid kidney stones, and renal disease (Edwards 2008). In the past few decades, gout has markedly increased in incidence and prevalence, and resulted in a significant degradation in the quality of life of patients (Saag et al. 2006; Wallace et al. 2004). To screen and evaluate the gouty therapeutic drugs, gouty mice models have been established by administering uricase inhibitor (Stavric et al. 1969). In most studies, uric acid supplements were also added to the diet, resulting in 6- to 10-fold increases in serum uric acid levels (Bradley et al. 1994). Targeted deletion of the uricase gene in mice also results in marked hyperuricemia, intrarenal urate crystal deposition, and renal failure (Thomas et al. 1985).

Although mice have been used for screen and evaluation of gouty therapeutic drugs for decades, the use of mice for the development of gouty therapeutic drugs creates a number of problems. One is the difference between human and mice in the end product of purine metabolism; the end product of purine metabolism varies among species (Wu et al. 1992). Most mammals excrete allantoin because they have an enzyme, urate oxidase (EC 1.7.3.3), which degrades the sparingly soluble uric acid to a more soluble allantoin. Urate oxidase was lost in humans and certain other primates by deleterious mutations in the urate oxidase gene (Beauchamp et al. 1994). The second problem is cost: experiments in specific-pathogen-free facilities, which are essential for the maintenance of experimental mice, are expensive. Third, ethical issues exist surrounding the use of mammalian animals for the development of medicine, which is regulated by laws in European countries (Kaito et al. 2007). It is harder than ever to use mammals for experiments because of modern animal rights and animal welfare laws.

In order to overcome these problems, identification and evaluation of the therapeutic effects of chemicals in an alternative animal model is desirable. The silkworm, *Bombyx mori* L. (Lepidoptera: Bombycidae), is an important model insect, and has the potential to serve as a large-scale drug screening system. *Bombyx mori* has been used as a model animal for the study of bacterial pathogenicity and therapeutic effects of antibiotics (Orihara et al. 2008), antiviral (Ishii et al. 2008) and immunostimulatory agents (Hamamoto et al. 2004), and pharmacokinetics (Hamamoto et al. 2004). Silkworms and humans do not express uricase, an enzyme that degrades uric acid to allantoin, and are similar in purine metabolism, since the end product is uric acid for both silkworms and humans (Hayashi 1960).

In this study, we performed quantitative measurement of the uric acid-lowering effects of allopurinol or sodium bicarbonate in *B. mori*. The results demonstrated that the effectiveness of oral administration of various gouty therapeutic drugs to silkworms is consistent with results for humans, suggesting that *B. mori* larvae can be used as an animal model for screening and evaluation of gouty therapeutic drugs.

**Figure 1.**
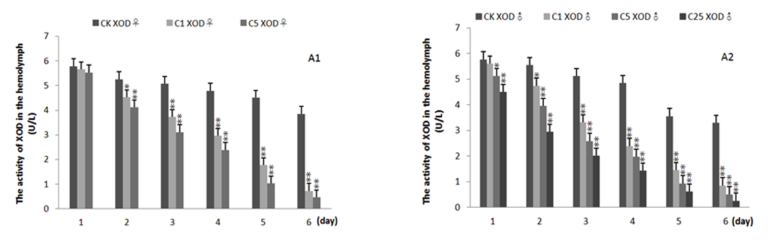
Effects of treatment of allopurinol on activity of xanthine oxidase in the hemolymph of 5^th^ instar *Bombyx mori*. A2 and A2 were for the female and male, respectively. CK: the control group, C1, C5, and C25 denoted the concentration of allopurinol were 1, 5, and 25 mg/mL, respectively (silkworm variety: Dazao, n = 5). High quality figures are available online.

## Materials and Methods

### Chemicals

Allopurinol and sodium bicarbonate were purchased from Jiangsu Fangqiang Phatmaceutical Ltd. (www.jsfqzy.com) and Stars Pharmaceutical Institute, respectively. The kits of xanthine oxidase (XOD) and uric acid (UA) were purchased from Nanjing Jiancheng Bioengineering Institute (www.njjcbio.com) and Mindray Bio-Medical Electronics Co. Ltd. (www.mindray.com), respectively.

### Silkworms

*Bombyx mori* (Dazao strain) were fed with fresh mulberry at 25 °C until they developed to the fifth instar larva. The silkworms used in our experiment were all age matched (from the 2^nd^ to the 8^th^ day of the fifth instar).

### Experimental design

On the first day of fifth instar larvae, the female and male silkworms were isolated and continuously fed on fresh mulberry leaves coated with allopurinol or sodium bicarbonate at different concentrations. The concentrations of allopurinol were 1, 5, and 25 mg/mL, respectively; the concentrations of sodium bicarbonate were 5, 25, and 50 mg/mL, respectively. The negative control was water.

### Determined of xanthine oxidase activity and uric acid content

Hemolymph (0.03 to 0.1 mL) and fat bodies of five silkworms were harvested at 24-hour intervals after silkworms were treated with agents and stored at -20 °C. The hemolymph and 10% of fat bodies homogenate were analyzed determine the activity of XOD and content of UA; the determination was carried out according to the illustration provided by the manufacturer.

## Results

### Effects of treatment of allopurinol on the activity of xanthine oxidase and uric acid content in silkworms

**Figure 2.**
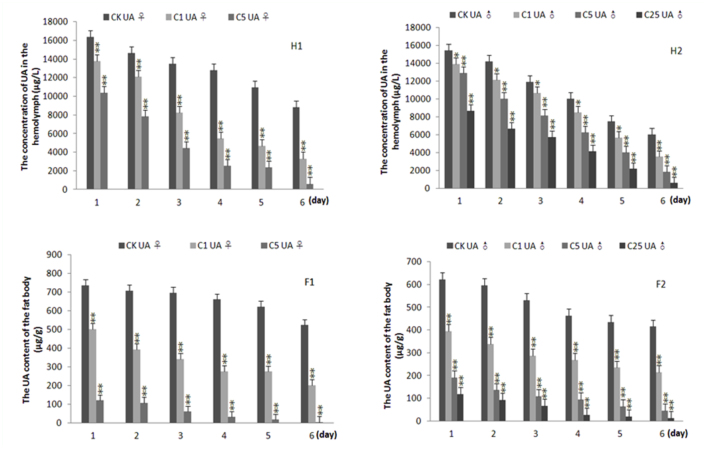
Effects of treatment of allopurinol on the content of uric acid in the hemolymph and fat body of 5^th^ instar *Bombyx mori*. H1 and H2 were for the content of uric acid in the hemolymph of the female and male, respectively. F1 and F2 were for the content of uric acid in the fat body of the female and male, respectively. CK: the control group, C1, C5, and C25 denoted the concentration of allopurinol were 1, 5, and 25 mg/mL, respectively (silkworm variety: Dazao, n = 5). High quality figures are available online.

Allopurinol is an inhibitor of XOD and was used as a gouty therapy drug in human. To explore whether the silkworm can be used as an animal model for identification and evaluation of gouty therapy, the activity of XOD in the hemolymph of female and male silkworms treated with allopurinol was investigated. The results are shown in Figure 1 and Figure 2. The activity of XOD in the hemolymph was reduced in the fifth instar silkworm. Compared with normal silkworms, the activity of XOD of silkworms treated with allopurinol was significantly lower than that of control silkworms, which were agematched in all experiments; moreover, the activity of XOD declined in a dose-dependent manner. The activity of XOD declined by 80% at six days post-treatment with allopurinol compared with that of the control silkworms.

The end product of purine metabolism is UA in silkworms. The content of UA in the hemolymph and fat body of silkworms were reduced in the fifth instar silkworm (Figure 2). Compared with the control silkworms, the concentration of UA in both the hemolymph and fat body of silkworms treated with allopurinol significantly declined in a dosedependent manner. The concentration of UA in both the hemolymph and fat body declined by 90 and 95%, respectively, at six days postadministration with 25 mg/mL of allopurinol (*p* < 0.01). These results indicate that the content of UA declined by inhibiting the activity of XOD.

**Figure 3.**
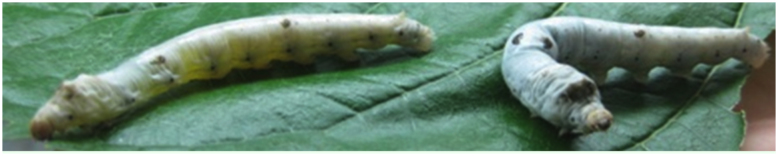
Appearance of 5^th^ instar *Bombyx mori* administered with allopurinol. Pictured is the silkworm treated with allopurinol (left) and the silkworm treated with H_2_O (right) (silkworm variety: Dazao). High quality figures are available online.

The epidermis of normal silkworms has a white color and opacity due to urate crystals deposited in the epidermis but the epidermis of silkworms treated with allopurinol became transparent (Figure 3). These results suggest that the effectiveness of treatments used to decrease UA content can be identified by the naked eye based on the transparency of the epidermis of silkworms.

**Figure 4.**
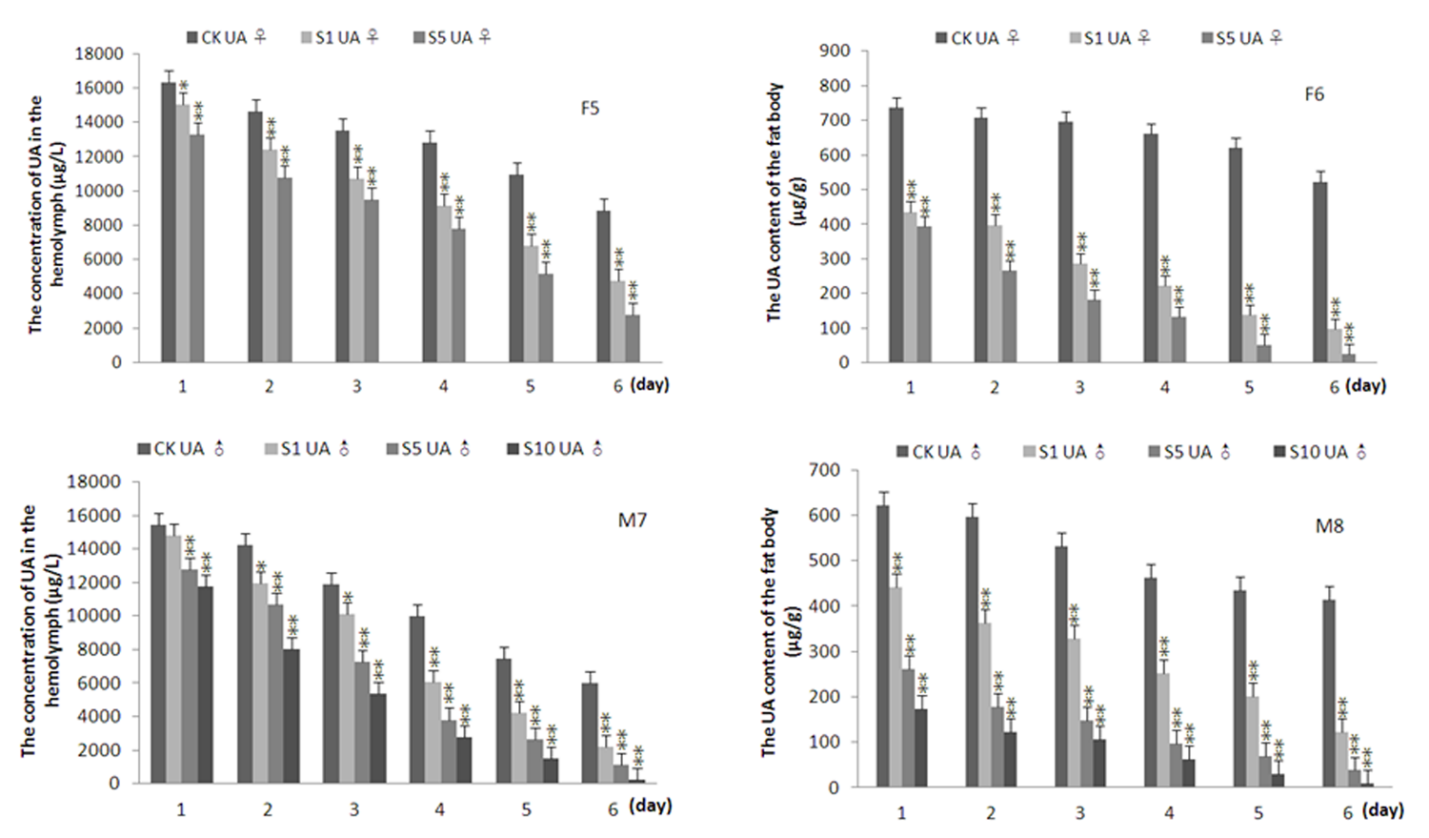
Effects of treatment of sodium bicarbonate on the content of uric acid in the hemolymph and fat body of 5^th^ instar *Bombyx mori*. F6 and F6 were for the content of uric acid in the hemolymph and fat body of the female, respectively. M6 and M6 was for the content of uric acid in the hemolymph and fat body of the male, respectively. CK denoted the control, S1, S5, and SlO* denoted the concentration of sodium bicarbonate were 5, 25, and 50 mg/mL, respectively (silkworm variety: Dazao, n = 5). High quality figures are available online.

### Effects of treatment of sodium bicarbonate on the activity of xanthine oxidase and uric acid content in silkworms

**Figure 5.**
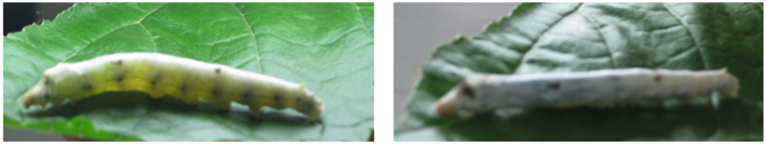
Appearance of the 5^th^ instar *Bombyx mori* administered with sodium bicarbonate. Pictured is the silkworm treated with sodium bicarbonate (left) and the silkworm treated with H_2_O (right) (silkworm variety: Dazao). High quality figures are available online.

To explore whether silkworms can be used as an animal model for identification and evaluation of gouty therapeutic drugs, silkworms were treated with sodium bicarbonate, a medicine for facilitating the excretion of UA via alkalization in humans. The results showed that treatment with sodium bicarbonate had no significant influence on the activity of XOD compared with the group fed with allopurinol (data not shown). However, the content of UA in the hemolymph and fat body of silkworms was reduced in a dose-depend manner compared with the control silkworms, which were agematched in all experiments (Figure 4). The epidermis of silkworms treated with sodium bicarbonate also became transparent (Figure 5). The concentration of UA in the both the hemolymph and fat body declined by 81 and 95%, respectively, at six days postadministration with 25 mg/mL of sodium bicarbonate (*p* < 0.01). These results indicated that the excretion of UA was facilitated by orally administering sodium bicarbonate.

## Discussion

Although mice have historically been used for identification and evaluation of drugs, problems with high cost, ethics, and biohazard dangers have made it desirable that the therapeutic effects of chemicals be identified and evaluated in an alternative animal model. Invertebrates have been used as an animal model of many diseases. Mahajan et al. (1999) investigated *Caenorhabditis elegans* as an animal model with the bacterium *Pseudomonas aeruginosa*, while Bernai et al. (2000) and Lemaitre et al. (1996) used the fruit fly *Drosophila melanogaster* as an animal model with *Escherichia coli*. Because these animals are too small to handle, however, they are not suitable for quantitative evaluation of therapeutic effects.

The complete sequence of the silkworm genome has been described (Xia et al. 2004). The silkworm is genetically tractable and many mutant lines have been constructed. Silkworms have several advantages as model animals for studying therapeutic effects of drugs. Despite differences in appearance, mice and silkworms are similar in ways that may allow them to be used interchangeably in study and evaluation of human drugs. Silkworms have analogous tissues/organs, they are large enough for hemolymph removal and analysis and for organs such as the midgut to be isolated, they have similar sensitivities to pathogens and comparable effects of drugs, and they are comparably low in cost, do not raise ethical problems, and display no biohazard dangers (Hamamoto et al. 2005).

Up to now, silkworm models have been used for assessing the therapeutic effects of chemicals, searching for immunostimulatory agents, detecting tests of poisons for drugs, and quantitatively evaluating therapeutic effects (Ishii et al. 2008; Hamamoto et al. 2004; Komto et al. 2004).

Silkworms and humans are similar in purine metabolism, since the end product of purine metabolism of both is UA (Hayashi 1960). In the silkworm, the main source of XOD is the fat body (Hayashi 1961). UA was distributed mainly in the fat body (Tojo 1971). UA is removed from the hemolymph and excreted with the meconium via the Malpighian tubules-hindgut system (Buckner et al. 1980). At different development stages, insects have different UA synthesizing capacity (USC) and urate excreting capacity. An 85% loss in USC occurs by the end of the feeding fifth larval instar of the tobacco hornworm, *Manduca sexta* (Williams-Boyce et al. 1980), as does the XOD activity (Buckner et al. 1993). The concentrations of UA in the hemolymph were lowest during the transition from the feeding stage to the wandering stage, at the time when there was a switch from UA excretion by the Malpighian tubule-hindgut system to storage in the fat body (Buckner et al. 1980). It was suggested that the UA storage in the fat body during the last larval instar of the tobacco hornworm is controlled by insect hormone (Buckner et al. 1980). In this study, we found that the UA level in the hemolymph and fat body decreased steadily during the feeding fifth larval instar of the silkworm, and was especially lowest at the end of the feeding fifth larval instar. This finding was similar to the results for tobacco hornworm, suggesting that the control of UA excretion and storage in the silkworm were similar to that in the tobacco hornworm.

XOD is an enzyme that catalyzes the oxidation of hypoxanthine to xanthine and can further catalyze the oxidation of xanthine to UA. The production of UA decreases by inhibiting the XOD activity with allopurinol, a gouty therapy drug used in humans. In this study, we found that the activity of XOD in the hemolymph of the silkworm fed mulberry leaves with allopurinol declined in a dosedependent manner, and was significantly lower than that of control silkworms. Moreover, the concentration of UA in the both the hemolymph and fat body also declined in a dose-dependent manner. These results indicate that the decrease of UA content in the silkworms treated with allopurinol was caused by the inhibition of XOD activity compared with that of the control, suggesting that silkworm larvae can be used as an animal model for screening and evaluation of gouty therapeutic drugs.

Sodium bicarbonate, is a gouty therapeutic drug for facilitating the excretion of UA via alkalization. In this study, we found that the content of UA in the hemolymph and fat body of silkworms decreased in a dose-depend manner in the treatment group fed with sodium bicarbonate, but the activity of XOD was not significantly lower than that of control silkworms, which are age matched in all experiments. This suggests that the excretion of UA in silkworm was facilitated by orally administering sodium bicarbonate. These results indicated that silkworm larvae can be used as an animal model for screening and evaluation of the drugs of UA excretion.

The epidermis of normal silkworms displays opaque skin due to urate crystals deposited in the epidermis. However, the epidermis of silkworms treated with allopurinol or sodium bicarbonate became transparent, which suggests that the effectiveness of treatments used to decrease UA content can be rapidly and conveniently judged according to the transparency of the epidermis of silkworms, and silkworm larvae can be used as an animal model for screening and evaluation of gouty therapeutic drugs. In addition, Normal silkworm larvae have opaque skin, while oil silkworm mutants have transparent or translucent skin owing to the lack of UA. More than 25 oily mutants of the silkworm were thought to be associated with the synthesis, transport, and accumulation of UA (Komto et al. 2004). Using oily mutants of the silkworm as animal models may be helpful for elucidating the mechanism of UA metabolism in other animals and developing therapeutic medicines of gout.

The silkworm is a domesticated insect and very sensitive to toxic substance. In this study, the development of silkworms orally administered with allopurinol or sodium bicarbonate was slightly delayed, the female silkworms fed mulberry leaves with 25 mg/mL of allopurinol or 50 mg/mL of sodium bicarbonate died successively, suggesting that silkworms can be used as a model for assessing the toxicity of drugs.

## Abbreviations

UA,: uric acid;
XOD,: xanthine oxidase
